# Patient Activation Among Individuals with Chronic Illness: A Cross-Sectional Study from Jordan

**DOI:** 10.3390/healthcare14020204

**Published:** 2026-01-13

**Authors:** Mohammad B. Nusair, Rawand Khasawneh, Fahad H. Baali, Ahmed B. Alkhalil, Samer A. Aldehoun, Sayer Al-Azzam

**Affiliations:** 1 Department of Clinical Pharmacy and Practice, Faculty of Pharmacy, Yarmouk University, Irbid 21163, Jordan; 2 Department of Clinical Pharmacy, Faculty of Pharmacy, Jordan University of Science and Technology, Irbid 22110, Jordan; rakhasawneh@just.edu.jo (R.K.); salazzam@just.edu.jo (S.A.-A.); 3 Department of Clinical Pharmacy, College of Pharmacy, Taif University, Taif 21944, Saudi Arabia; f.baali@tu.edu.sa

**Keywords:** patient activation, health literacy, polypharmacy, Jordan, chronic illness

## Abstract

**Background:** Managing chronic conditions can overwhelm patients and reduce their confidence in self-management. Patient activation (PA) is a concept that reflects patients’ knowledge, skills, and confidence in managing their health and self-care. In Jordan, PA has not been explicitly studied, particularly among patients with chronic conditions. Therefore, this study explores PA and its determinants in individuals with chronic conditions in Jordan. **Methods**: A cross-sectional study was conducted using a convenience sample of outpatients recruited from a tertiary hospital in Jordan. Participants completed a questionnaire including sociodemographic and clinical data, the Single Item Literacy Screener, and the 13-item Patient Activation Measure (PAM). Bivariate and regression analyses were conducted to explore the factors associated with PAM scores. **Results:** Among a total of 666 participants, the mean PAM score was 57.1 ± 9.17, indicating a moderate activation level overall. Regression analysis revealed that being female (*p* = 0.14), adequate health literacy (*p* = 0.002), and post-secondary education (*p* = 0.004) were significantly associated with higher PAM scores, and older age (*p* = 0.004) and polypharmacy (*p* = 0.010) with lower scores. An additional regression model showed that the negative association between polypharmacy and PA scores did not differ by health literacy level, with no significant interaction between polypharmacy and health literacy (*p* = 0.555). **Conclusions:** This study showed that individuals with chronic illnesses in Jordan had moderate to high patient activation levels. Several sociodemographic and clinical factors were significantly associated with patient activation. Polypharmacy was independently associated with lower patient activation scores, regardless of health literacy levels. However, given the study’s exploratory nature, the results should be interpreted as preliminary evidence warranting further research.

## 1. Introduction

The number of individuals living with multiple chronic conditions is steadily rising, posing a growing challenge to healthcare systems worldwide [1]. Managing multiple chronic conditions substantially burdens patients, contributing to complex care needs, frequent healthcare utilization, and diminished quality of life [2,3]. Moreover, people with multiple chronic conditions are more susceptible to polypharmacy (i.e., the concomitant use of five medications or more) [4,5], which increases the likelihood of adverse drug reactions, medication nonadherence, and functional decline [6]. The complexity of managing multiple medications and chronic conditions can be overwhelming, diminishing patients’ confidence and ability to manage their health effectively [7,8].

Patient activation (PA), a construct that evaluates patients’ knowledge, skills, and confidence in managing their health and healthcare decisions, is a cornerstone of patient-centered care [9]. PA is closely related to health literacy, defined as the ability to obtain, understand, and process basic health information required to make appropriate health-related decisions [10]. PA reflects an individual’s ability in self-management behaviors and preventive care [11]. Self-management behaviors for individuals living with chronic conditions include medical management tasks (e.g., attending medical appointments, adhering to treatment plans, and self-assessment for complications), behavioral changes (e.g., balanced diet, exercise, and creating medication routines), and emotional management (e.g., managing the psychological consequences of living with a chronic condition) [7]. Evidence suggests that increased PA is associated with improvements in self-management behaviors, such as exercise and stress management, which in turn are linked to better healthcare outcomes [7,12]. PA is also associated with better medication adherence, as patients with a greater understanding of their conditions and treatments are more likely to take medications as prescribed and recognize early signs of adverse effects [9]. Moreover, a recent meta-analysis found that high PA scores were associated with fewer emergency department visits, fewer hospitalizations, and reduced healthcare utilization [13]. Further, higher PA levels correlate with reduced medication-related complications [14].

Despite the well-established advantages of PA for individuals living with chronic conditions, healthcare in resource-limited settings often remains prescriber-driven, consigning patients to passive recipients [15,16]. This traditional model can lead to inadequate self-care, ineffective medication use, poor patient health outcomes, and increased healthcare costs [11]. For patients with polypharmacy, this may be particularly overwhelming and may undermine their ability to engage in effective self-management behaviors. This is explained by the cognitive load theory, as managing complex medication regimens increases cognitive demand on patients and may ultimately reduce their engagement in self-management practices and activation [17]. Similarly, there is clear evidence that low health literacy is associated with increased cognitive demands and low PA [18,19]. However, it’s unclear if polypharmacy and low health literacy have an independent effect on PA rather than a synergetic effect.

In Jordan, over 30% of adults live with at least one chronic medical condition [20,21], and polypharmacy is reported to be prevalent (47.5%) among adults with chronic conditions [22,23,24]. Patient-centered practice in the Middle East and North Africa (MENA) region, including Jordan, is limited and predominantly prescriber-driven. This may lead to low PA and ineffective disease and medication management, especially in patients with chronic illnesses and polypharmacy [25]. Previous research has established a relationship between polypharmacy and poor medication management and outcomes [6]. However, the relationship between polypharmacy and PA remains underexplored in low- and middle-income countries, particularly in the MENA region. Therefore, this study aimed to explore PA and its relationship with polypharmacy among individuals with chronic illness in Jordan, contributing to the limited literature in these areas with the following specific objectives: (1) investigate the levels of PA among adults with chronic diseases in Jordan; (2) explore the associations between PA and sociodemographic and clinical factors; and (3) explore the association between PA and polypharmacy.

## 2. Materials and Methods

### 2.1. Study Design and Setting

This was a cross-sectional study of a convenience sample of outpatients recruited from clinics at King Abdullah University Hospital (KAUH), a teaching hospital in Jordan. This study was approved by the Institutional Review Board (IRB) at Yarmouk University (Reference no: IRB/2024/648).

### 2.2. Participants

Participants were recruited from November 2024 through January 2025 at KAUH outpatient clinics and the outpatient pharmacy. A trained clinical pharmacist screened patients at these sites and invited those who met the inclusion criteria to take part in this study. The clinical pharmacist provided patients with information about the study’s aims and voluntary nature. The inclusion criteria were (1) being aged 18 years or over and (2) having at least one chronic condition (i.e., a medical condition that is expected to last 12 months or more and requires ongoing medical attention or limits activities of daily living). Eligible participants were required to self-report at least one diagnosed chronic condition confirmed through their medical records. Patients showing any signs of moderate or severe cognitive impairment were excluded from this study. After providing informed consent, participants were interviewed by a clinical pharmacist and completed a questionnaire that consisted of three sections.

### 2.3. Data Sources

Data were collected using a structured, interviewer-administered questionnaire comprising the following components:

(1) sociodemographic and clinical variables, including age, gender, insurance, employment status, income, marital status, number of medications, and chronic medical conditions. Participants were asked to report all medications they had taken regularly in the past four weeks, including prescription and nonprescription medications and supplements. Polypharmacy was defined as the concurrent use of five or more medications.
(2) the Single Item Literacy Screener (SILS) was used to assess functional health literacy related to reading and understanding written health information [26]. Patients report how often they require assistance reading and understanding health information and instructions from healthcare providers using a 5-point Likert scale (1: never to 5: always). A score of 3–5 (i.e., sometimes, often, or always) indicates limited health literacy, whereas a score of 1–2 (i.e., never or rarely) indicates adequate health literacy [26]. In this study, we used the Arabic version of the SILS [27]. The SILS has strong evidence of validity and correlates with more complex health literacy assessments (e.g., S-TOFLHA) [26,28,29,30].
(3) the Patient Activation Measure-13 (PAM), a widely recognized tool for assessing patient knowledge, skills, and confidence in self-management. The PAM has undergone extensive validation in over 30 languages and countries and has been used in more than 800 published research studies [31]. Patients’ responses to the PAM are converted into activation scores and levels using a computer algorithm provided by Insignia Health, LLC. (Portland, OR, USA). Patient activation scores range from 0 to 100, with higher scores indicating greater activation. PAM scores categorize patients into four levels: level one: individuals lack confidence and are passive, disengaged, and overwhelmed; level two: individuals have some healthcare knowledge but are still struggling; level three: individuals are building self-management skills and gaining control; and level four: individuals have adopted new behaviors and aim to maintain a healthy lifestyle and positive health behaviors but with some challenges in maintaining them [32,33].

All data collection tools (i.e., Arabic versions of PAM-13 and SILS) have been previously used with Arabic-speaking participants and have demonstrated strong validity and reliability; therefore, the pilot study was deemed unnecessary.

### 2.4. Study Size

Raosoft was used to calculate the minimum required sample size [34]. The estimated number of adults in Jordan (i.e., aged 18 years or over) living with at least one chronic condition is 2.4 million. Therefore, based on a 95% confidence interval (CI) and a 5% margin of error, the minimum required sample size was 385 participants.

### 2.5. Statistical Methods

All statistical analyses were conducted using IBM SPSS Statistics (SPSS Inc., Chicago, IL, USA) version 28. Descriptive statistics were used to summarize participants’ sociodemographic and clinical characteristics. Categorical variables were presented as frequencies and percentages, while continuous variables were summarized using means and standard deviations (SD). PAM scores and levels were calculated using a computer algorithm provided by Insignia Health. Responses to the PAM were analyzed both continuously (i.e., PAM score, range 0–100) and categorically (i.e., based on the four standardized activation levels).

Bivariate analyses were performed to assess the associations between the independent variables and patient activation. PAM scores did not meet the normality assumption according to the Shapiro–Wilk and Kolmogorov–Smirnov tests (*p* < 0.001); therefore, nonparametric analyses were used. Chi-square tests were used to evaluate associations between the independent variables and patient activation level (i.e., a categorical variable), and the Mann–Whitney U test was used to evaluate associations between the independent variables and patient activation score (i.e., a continuous variable). For independent variables with more than two groups, the Kruskal–Wallis test was used, followed by the Bonferroni–Dunn post hoc test to identify differences between pairs of groups. Spearman’s rank-order correlation was calculated to evaluate the relationship among the continuous variables (i.e., age, number of medications, and number of chronic conditions).

A multiple linear regression model was used to identify variables significantly associated with patient activation, with PAM score as the dependent variable. The independent variables entered into the model were polypharmacy (i.e., yes/no), health literacy (i.e., adequate/limited), number of medications, number of chronic conditions, age, income, gender, educational level, and employment status. Statistical significance was set at *p* < 0.05, and all tests were two-tailed.

Further, an interaction term (Polypharmacy × Health Literacy) was created and included in the model to examine whether the effect of polypharmacy on activation was moderated by health literacy. An estimated marginal means plot was generated to visualize the interaction between health literacy and polypharmacy on patient activation scores.

## 3. Results

A total of 666 patients participated in this study. The mean age of participants was 55.7 ± 15.68 years, and the majority were female (n = 396, 59.5%). Of the 666 participants, 60.4% had adequate health literacy, and 52% were polypharmacy patients. The average number of medical conditions per patient was 2.5 ± 1.51, with an average of 5.3 ± 3.38 medications per patient (Table 1).

### 3.1. PAM Results

Participants had a mean PAM score of 57.1 ± 9.17 (min: 29, max: 100), indicating a moderate activation level overall. The majority of participants (58.6%) fell into levels three and four, while 41.5% fell into levels 1 and 2 (Supplementary Materials, Table S1). The Mann–Whitney U test indicated that female participants, those with post-secondary education, and those who were employed all demonstrated significantly higher activation levels and scores compared to their respective counterparts (*p* < 0.001, *p* < 0.001, *p* = 0.006, respectively). The Kruskal–Wallis test indicated a significant difference in PAM scores across monthly income groups (Table 1). Moreover, participants with adequate health literacy had significantly higher levels of patient activation and scores (*p* < 0.001) compared to participants with limited literacy, while polypharmacy participants had significantly lower levels of patient activation and scores compared to non-polypharmacy participants (*p* < 0.001, Table 1 and Table S1). Furthermore, Spearman’s correlation test indicated that patient activation scores were negatively correlated with age, the number of medications, and the number of medical conditions (*p* < 0.001; Table 1).

### 3.2. Factors Associated with Patient Activation

To identify variables significantly associated with patient activation scores, a multiple linear regression was conducted. The overall model was statistically significant (F = 13.567, *p* < 0.001), indicating that the included variables jointly predicted patient activation scores better than chance. The regression model accounted for approximately 11.8% of the variance in patient activation scores (R^2^ = 0.118; adjusted R^2^ = 0.108), reflecting modest explanatory power and suggesting that additional unmeasured factors may contribute to patient activation. The results of the multiple linear regression showed multiple factors were significantly associated with PAM scores (Table 2). Being female, adequate health literacy, and higher educational attainment were associated with increased patient activation scores (B = 1.741, B = 2.336, B = 2.083, respectively). Polypharmacy was significantly associated with lower activation scores, whereby patients with polypharmacy had PAM scores of 1.90 points lower than those without polypharmacy. Similarly, age was negatively associated with patient activation scores, with each additional year of age linked to a 0.069-point decrease in PAM score (Table 2).

**Figure 1:**
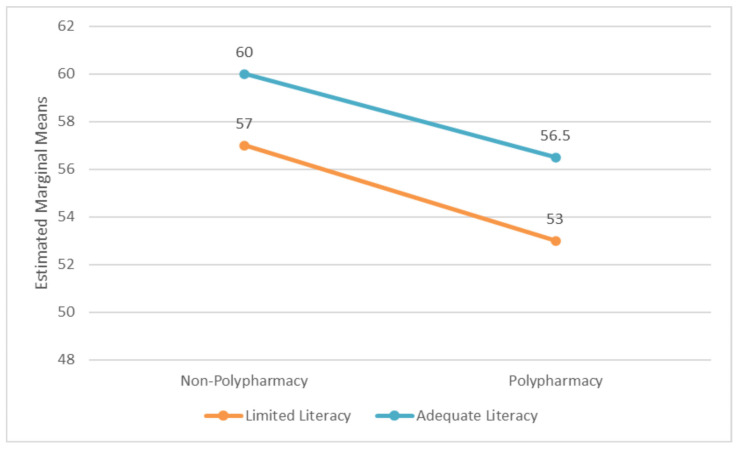
Estimated marginal means of patient activation score by health literacy and polypharmacy.

A further multiple linear regression was performed to assess the impact of polypharmacy and health literacy on patient activation and the potential interaction between the two (polypharmacy × health literacy). The model showed that polypharmacy was significantly associated with lower patient activation scores, while health literacy was not statistically significant with the presence of the interaction variable (Table 3). The interaction between polypharmacy and health literacy was not statistically significant (B = 0.854, *p* = 0.555), suggesting that polypharmacy does not affect patient activation scores differently across health literacy levels. Figure 1 shows that participants with adequate or limited health literacy have lower patient activation scores when they have polypharmacy. The lines in the figure are nearly parallel, suggesting that the effects of polypharmacy and health literacy on patient activation are independent and additive but not synergistic.

## 4. Discussion

This study aimed to investigate the levels and factors associated with patient activation among adults with chronic diseases in Jordan, as well as the role of health literacy and polypharmacy. The results showed that 58.6% of the participants had moderate or high patient activation levels, while the remaining participants (41.5%) fell into lower activation levels (i.e., levels 1 and 2).

The mean PAM score in our study (57.1 ± 9.17) is comparable to some studies in other countries, which have also reported moderate levels of patient activation. For example, a study in Singapore investigated patient activation among patients with chronic diseases and reported a mean PAM score of 58.57 ± 10.79 [35], while another study among Dutch diabetic patients reported a mean PAM score of 57.4 ± 14.3 [36]. However, it remains lower than the average patient activation scores, which range from 59.4 to 74.1 [37,38,39,40]. This is a surprising finding given that previous studies in Jordan have reported that individuals with chronic illnesses have high self-efficacy, high motivation, and good self-care practices [41,42]. Multiple factors could contribute to the variation in global findings on patient activation, including patient-related factors (e.g., patient characteristics and sociodemographic factors), disease-related factors (e.g., type of chronic illness and the complexity of disease management), and healthcare system-related factors (e.g., patient-centeredness and shared decision-making) [39].

The findings of this study showed patient activation to be significantly associated with gender, age, educational level, health literacy, and polypharmacy (each with a modest effect), indicating that patient activation has a multifactorial nature and is shaped by the cumulative effect of sociodemographic and clinical factors rather than a single factor. Being female was associated with modestly higher patient activation scores, which correlates with the findings of previous studies that female patients are significantly more likely than male patients to seek medical advice, adhere to preventive measures, and respond to health-behavioral changes [43,44,45]. In Jordan and many MENA societies, women often assume caregiving roles within families, including medication management, appointment scheduling, and disease monitoring for themselves and others. Such a socially constructed role may contribute to stronger health navigation skills and better health management, ultimately leading to better patient activation [46].

Older age was associated with slightly lower patient activation scores, with the effect size suggesting a gradual decline in activation with aging. Our finding is also consistent with previous studies that have reported older age to be associated with higher comorbidity, contributing to lower activation [43,47,48]. Older patients have also been reported to be less confident in maintaining health behaviors and more accustomed to paternalistic and traditional hierarchical healthcare beliefs [48,49,50]. Moreover, our study found higher educational attainment to be associated with modestly higher patient activation. Higher education contributes to better knowledge and self-efficacy in self-care and health management, ultimately leading to higher activation [43,50,51,52]. Furthermore, functional health literacy was significantly associated with higher patient activation, although the magnitude of this association was modest and may underestimate the effect of the interactive and critical domains of health literacy. This finding also aligns with several studies in the literature [8,50,53]. Individuals with limited health literacy are more likely to be passive in shared decision-making, feel overwhelmed, and exhibit inadequate or limited self-care behaviors [50,54,55,56]. In Jordan and many MENA contexts, lower education and health literacy, coupled with patients’ culturally rooted deference to physicians and expectation of physician-led decision-making, can contribute to lower patient activation [57]. Such a paternalistic care model may limit patients’ perceived role in self-management even when access to healthcare is adequate. The findings also indicated that polypharmacy was modestly associated with low patient activation, with a comparable magnitude to other determinants. A similar association was reported by Ge et al. [58]. Polypharmacy increases the risk of drug interactions and adverse reactions, which can lead to fear of taking medications, poor adherence, and reluctance to manage one’s own health [59,60]. Moreover, evidence suggests that polypharmacy is linked to cognitive impairment, which may confuse patients in medication management and active participation in their own health care [60,61]. In Jordan and many MENA regions with similar healthcare systems, patients often lack a single point of reference and receive medications from multiple providers. In addition to this, medication reconciliation and structured deprescribing are not routinely implemented. Thus, lower patient activation levels in polypharmacy patients may correlate to system complexity rather than disease severity alone in such settings [25]. Such complexity can lead to confusion, fear of adverse effects, and reduced confidence in self-management.

Polypharmacy, like other determinants identified in this study, showed a small effect size on patient activation (i.e., an average reduction of 1.9 points in the PAM score) and may not, alone, have significant clinical implications. However, the presence of these determinants combined could produce a significant change in activation levels and clinical implications, as patient activation is a multifactorial construct.

Moreover, the regression model explained a relatively small proportion of the variance in patient activation, suggesting that it could be influenced by other determinants not assessed in this study. Factors such as disease-specific burden, quality of chronic illness care, self-efficacy, motivation, interactive and critical domains of healthy literacy, and social support may play important roles [18,62]. Therefore, the regression findings should be interpreted as exploratory associations rather than comprehensive explanations of patient activation.

According to cognitive load theory, both polypharmacy and low health literacy can increase cognitive demand and require greater mental effort during chronic disease self-management, potentially negatively influencing patient activation [17,18,19]. To explore whether these two factors have an additive or synergistic effect on patient activation, we conducted a regression analysis that included polypharmacy, health literacy, and their interaction (polypharmacy × health literacy) as predictors, with PAM score as the dependent variable. The regression analysis indicated no significant interaction effect, suggesting that polypharmacy and health literacy influence patient activation independently. However, using SILS as a proxy for health literacy may have influenced the interaction effect, as SILS does not assess the interactive and critical domains of health literacy, which may contribute to cognitive overload. Therefore, future studies are needed to investigate the interaction effect employing all domains of the health literacy construct.

Given the exploratory nature of the study, the provided recommendations focus on awareness and potential areas for improvement rather than definitive policy mandates. At the level of practice, the study findings have significant implications for healthcare professionals (HCPs) and patients. Healthcare professionals should adopt a patient-centered approach that involves patients in decision-making, empowers them, and enhances their understanding and engagement. As for polypharmacy patients, clinicians are advised to follow strategies, such as medication review or deprescribing, to reduce treatment burden as clinically applicable. Such approaches are expected to improve patient engagement and confidence in self-management and should be envisioned as supportive measures rather than universal solutions. Additionally, educational interventions, such as the provision of clear and tailored education about medications, disease management, and self-care, may help address barriers to patient engagement, especially among patients with complex regimens. Notably, the effectiveness of such strategies requires further evaluation through longitudinal or interventional studies. Finally, technology-based tools (such as patient reminders or telehealth services) may offer additional support for patient engagement.

### Strengths and Limitations

To our knowledge, this study is the first to explore patient activation among individuals with chronic illnesses in Jordan and the MENA region. This study also contributes to the limited literature on the association between patient activation and polypharmacy. However, this study has several methodological weaknesses that may limit the robustness and generalizability of the findings.

One important limitation of this study is that participants were recruited through convenience sampling at a single tertiary hospital. This introduces a risk of selection bias, whereby more health-conscious and engaged individuals are more likely to participate, leading to an overestimation of patient activation levels. Additionally, this limits the generalizability of the findings, as our sample may not reflect the broader population of individuals living with chronic conditions in Jordan.

Another key limitation is that health literacy was assessed using the SILS, which is a brief tool designed to identify perceived difficulty with reading health-related materials. While this tool has demonstrated validity as a screening measure of the functional domain of health literacy, it does not assess the interactive and critical domains [26]. These dimensions may differentially influence patients’ ability to engage in shared decision-making and manage complex treatment regimens. Therefore, the restricted scope of the SILS may have attenuated the observed associations involving health literacy, and more comprehensive assessments may be needed to capture the full complexity of health literacy in relation to patient activation.

The absence of disease-specific data is another key limitation of this study, as chronic diseases differ in terms of burden, treatment complexity, and self-management needs, all of which may influence patient activation. Consequently, this limited the ability to assess heterogeneity within the sample, examine patient activation across different disease types or complexity, and compare findings with disease-specific patient activation literature. The lack of disease-specific data limits the applicability and generalizability of the findings to a specific chronic disease group.

## 5. Conclusions

This study showed that individuals with chronic illnesses in Jordan had moderate to high patient activation levels. Gender, age, educational level, health literacy, and polypharmacy were associated with patient activation. Notably, polypharmacy was independently associated with lower patient activation scores, regardless of health literacy levels. In light of these findings, integrating patient-centered practices, shared decision-making, and medication management strategies may help empower patients with chronic diseases and increase their activation levels, particularly among those with complex medication regimens. This study addresses an important and underexplored topic; however, given its exploratory nature, modest explanatory power, and limitations, the findings should be interpreted as preliminary evidence warranting further research.

## Figures and Tables

**Table 1:** Patient Activation Measure (PAM) Scores by Sociodemographic Characteristics and Clinical Variables.

Variable		n (%)	PAM Score (Mean ± SD)	*p* Value
Gender	Female	396 (59.5%)	58.1 ± 8.75	<0.001
	Male	270 (40.5%)	55.7 ± 9.59	
Marital Status	Single/never married	59 (8.9%)	57.4 ± 9.60	0.948
	Married/Previously married	607 (91.1%)	57.0 ± 9.12	
Education	Up to secondary education	360 (54.1%)	55.5 ± 8.73	<0.001
	Post-secondary education	306 (45.9%)	59.0 ± 9.31	
Insurance	Insured	497 (74.6%)	57.3 ± 9.25	0.251
	No insurance	169 (25.4%)	56.4 ± 8.90	
Employment Status	Employed	150 (22.5%)	58.9 ± 9.62	0.006
	Unemployed/Retired	516 (77.5%)	56.5 ± 8.96	
Monthly Income	<500 JOD	362 (54.4%)	55.7 ± 8.74	0.001 *
	500–999 JOD	260 (39%)	58.1 ± 8.77	
	1000–1499 JOD	30 (4.5%)	61.4 ± 12.5	
	1500 JOD or more	14 (2.1%)	64 ± 11.17	
Health Literacy	Adequate	402 (60.4%)	58.7 ± 8.78	<0.001
	Limited	263 (39.6%)	54.7 ± 9.22	
Polypharmacy	Yes	346 (52%)	55.2 ± 8.71	<0.001
	No	320 (48%)	59.1 ± 9.24	
Age (mean ± SD), Spearman’s correlation (*ρ*)		*ρ* = −0.232	55.7 ± 15.68	<0.001
Number of chronic medical conditions per patient (mean ± SD), Spearman’s correlation (*ρ*)		*ρ* = −0.152	2.5 ± 1.51	<0.001
Number of medications per patient (mean ± SD), Spearman’s correlation (*ρ*)		*ρ* = −0.186	5.3 ± 3.38	<0.001

* Based on the Kruskal–Wallis test. The Bonferroni–Dunn post hoc test showed a significant difference between the <500 JOD group vs. the 999 JOD group, and the <500 JOD vs. the ≥1500 JOD group.

**Table 2:** Linear Regression Analysis of Factors Associated with Patient Activation Scores.

Variable	B	SE	β	*p*-Value	95% CI
Polypharmacy (Yes)	−1.900	0.735	−0.104	0.010	[−3.344, −0.456]
Health Literacy (Adequate)	2.336	0.737	0.125	0.002	[0.888, 3.785]
Age (years)	−0.069	0.024	−0.118	0.004	[−0.117, −0.022]
Gender (Female)	1.741	0.706	0.093	0.014	[0.355, 3.126]
Education Level (Post-secondary education)	2.083	0.721	0.113	0.004	[0.668, 3.499]

**Table 3:** Linear Regression Examining the Interaction Between Polypharmacy and Health Literacy on Patient Activation.

Variable	B	SE	β	*p*-Value	95% CI
Polypharmacy (Yes)	−3.674	1.142	−0.200	0.001	[−5.916, −1.433]
Health Literacy (Adequate)	1.977	2.381	0.106	0.407	[−2.699, 6.652]
Interaction: Polypharmacy × Health Literacy	0.854	1.447	0.075	0.555	[−1.988, 3.696]

Note: The dependent variable is PAM Score (range 0–100). The interaction term represents the combined effect of polypharmacy and health literacy.

## Data Availability

The data presented in this study are available on request from the corresponding author. Data are not publicly available due to privacy and ethical restrictions.

## Supplementary Materials

The following supporting information can be downloaded at: https://www.mdpi.com/article/10.3390/healthcare14020204/s1, Table S1: Distribution of Patient Activation Measure (PAM) Levels Across Participant Characteristics.

## Abbreviations

The following abbreviations are used in this manuscript:

PA	Patient Activation
PAM	Patient Activation Measure
MENA	Middle East and North Africa
