# Supplementary material for: Patient Activation Among Individuals with Chronic Illness: A Cross-Sectional Study from Jordan
Source: Healthcare (Basel). 2026 Jan 13;14(2):204. doi: 10.3390/healthcare14020204 (PMC12840788; doi:10.3390/healthcare14020204)
Supplement: Supplementary file 1 [file healthcare-14-00204-s001.zip › healthcare-4037432-supplementary.pdf]

Table S1. Distribution of Patient Activation Measure (PAM) Levels Across Participant Characteristics.

Variable		n (%)	PAM Level 1 (n=65) n (%)	PAM Level 2 (n=211) n (%)	PAM Level 3 (n=350) n (%)	PAM Level 4 (n=40) n (%)	P value
Gender	Female	396 (59.5%)	26 (6.6%)	115 (29.0%)	231 (58.3%)	24 (6.1%)	<.001
	Male	270 (40.5%)	39 (14.4%)	96 (35.6%)	119 (44.1%)	16 (5.9%)	
Marital Status	Single/never married	59 (8.9%)	4 (6.8%)	21 (35.6%)	29 (49.2%)	5 (8.5%)	.637
	Married/ Previously married	607 (91.1%)	61 (10.0%)	190 (31.3%)	321 (52.9%)	35 (5.8%)	
Education	Up to secondary education	360 (54.1%)	43 (11.9%)	130 (36.1%)	173 (48.1%)	14 (3.9%)	.001
	Post-secondary education	306 (45.9%)	22 (7.2%)	81 (26.5%)	177 (57.8%)	26 (8.5%)	
Insurance	Insured	497 (74.6%)	48 (9.7%)	152 (30.6%)	266 (53.5%)	31 (6.2%)	.731
	No insurance	169 (25.4%)	17 (10.1%)	59 (34.9%)	84 (49.7%)	9 (5.3%)	
Employment Status	Employed	150 (22.5%)	12 (8.0%)	38 (25.3%)	89 (59.3%)	11 (7.3%)	.149
	Unemployed/ Retired	516 (77.5%)	53 (10.3%)	173 (33.5%)	261 (50.6%)	29 (5.6%)	
Monthly Income	< 500 JOD	362 (54.4%)	42 (11.6%)	129 (35.6%)	176 (48.6%)	15 (4.1%)	.003
	500-999 JOD	260 (39%)	19 (7.3%)	72 (27.7%)	151 (58.1%)	18 (6.9%)	
	1000-1499 JOD	30 (4.5%)	3 (10.0%)	8 (26.7%)	16 (53.3%)	3 (10.0%)	
	1500 JOD or more	14 (2.1%)	1 (7.1%)	2 (14.3%)	7 (50.0%)	4 (28.6%)	
Health Literacy	Adequate	402 (60.4%)	23 (5.7%)	121 (30.1%)	228 (56.7%)	30 (7.5%)	<.001
	Limited	263 (39.6%)	42 (15.9%)	90 (34.1%)	122 (46.2%)	10 (3.8%)	
Polypharmacy	Yes	346 (52%)	42 (12.1%)	125 (36.1%)	168 (48.6%)	11 (3.2%)	<.001
	No	320 (48%)	23 (7.2%)	86 (26.9%)	182 (56.9%)	29 (9.1%)	
